# Blood Based Biomarkers for Predicting Treatment Response to Immune Checkpoint Inhibitors After EGFR‐TKI Resistance in Non‐Small Cell Lung Cancer

**DOI:** 10.1111/1759-7714.70257

**Published:** 2026-03-13

**Authors:** Min Seok Park, Jun Hyeok Lim, Nuri Park, Eunji Park, Ayoung Lim, Suji Lee, Yejin Cho, Sehan Kwak, Minseo Lee, Donghyun Seo, Lucia Kim, Woo Kyung Ryu, Jeong‐Seon Ryu, Eun Young Kim, Soon‐Sun Hong, Kyung Hee Jung

**Affiliations:** ^1^ Department of Biomedical Sciences College of Medicine, Inha University Incheon Republic of Korea; ^2^ Division of Pulmonology, Department of Internal Medicine Inha University Hospital, Inha University College of Medicine Incheon Republic of Korea; ^3^ Yonsei University College of Medicine Seoul Republic of Korea; ^4^ Department of Medicine Inha University College of Medicine Incheon Republic of Korea; ^5^ Department of Pathology Inha University Hospital, Inha University College of Medicine Incheon Republic of Korea; ^6^ Division of Pulmonary and Critical Care Medicine, Department of Internal Medicine Yonsei University College of Medicine Seoul Republic of Korea

**Keywords:** epidermal growth factor receptor, immune checkpoint inhibitor, liquid biopsy, non‐small cell lung cancer, predictive biomarker, treatment response, tyrosine kinase inhibitor

## Abstract

**Background:**

Immune checkpoint inhibitors (ICIs) have limited benefit in epidermal growth factor receptor (EGFR)‐mutant non‐small cell lung cancer (NSCLC). However, they are often tried after tumors develop resistance to EGFR tyrosine kinase inhibitors (TKIs). Because EGFR‐TKI treatment alters the tumor microenvironment, biomarkers predictive of ICI response are ideally identified post‐EGFR‐TKI resistance, but obtaining repeat biopsies at this time can be challenging. The purpose of this study was to explore predictive biomarkers for ICI response using plasma samples collected after EGFR‐TKI therapy.

**Methods:**

This retrospective analysis included 28 patients with EGFR‐mutant NSCLC treated with an ICI after developing resistance to EGFR‐TKI. Plasma samples collected at TKI progression were profiled using an Olink Target 96 immune protein panel to identify differential protein expression. Candidate protein biomarkers were validated by immunohistochemistry in tumor tissue. Durable clinical response (DCB) was defined as patients achieving progression‐free survival (PFS) ≥ 6 months during ICI therapy.

**Results:**

Of the 28 patients, 6 (21.4%) achieved durable clinical benefit, with PFS ≥ 6 months. Proteomic analysis identified four plasma proteins that differed significantly between DCB and NCB. Gal‐9 and GZMH levels were elevated in NCB, whereas IL‐4 and IL‐6 were elevated in DCB. Notably, PFS was significantly longer in patients with lower Gal‐9 and higher IL‐4 levels.

**Conclusions:**

Plasma‐based immune markers measured at the time of TKI resistance may help predict which patients with EGFR‐mutant NSCLC will respond to subsequent ICI therapy. Such biomarkers could guide immunotherapy decision‐making in this clinically challenging population.

## Graphical abstract

In EGFR‐mutant non‐small cell lung cancer, immune checkpoint inhibitors generally show limited efficacy. By integrating plasma proteomics and tumor immunohistochemistry, we identified biomarkers associated with durable clinical benefit and progression‐free survival following immunotherapy. These results provide a potential predictive framework for patient stratification in this biologically distinct population.

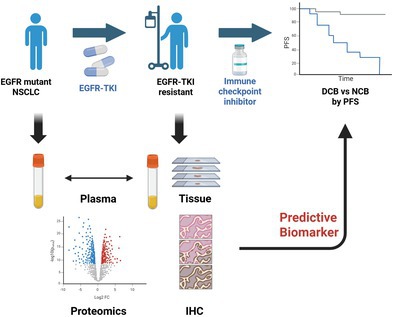

## 1 Introduction

Epidermal growth factor receptor (EGFR)‐mutant non‐small cell lung cancer (NSCLC) typically responds well to first‐line EGFR tyrosine kinase inhibitors (TKIs), but acquired resistance to TKI therapy inevitably develops [1]. After TKI failure, treatment options are limited, and immune checkpoint inhibitors (ICIs) are often used, despite historically poor efficacy in this subset of tumors. This lack of ICI benefit is mainly attributed to an “immunologically cold” tumor microenvironment in EGFR‐driven tumors, characterized by low tumor mutational burden, low programmed cell death ligand 1 (PD‐L1) expression, and sparse T‐cell infiltration [2, 3]. Adding ICIs after the development of TKI resistance in EGFR‐mutant tumors failed to improve outcomes in recent phase III trials [4, 5]. Nevertheless, occasional responses to ICIs are observed in EGFR‐mutant NSCLC, and identifying predictors of these beneficial effects remains an important clinical issue [6].

Because ICIs are typically administered after TKI failure, any predictive biomarker should ideally be assessed on samples obtained at the time of acquired TKI resistance. Emerging evidence suggests that the tumor immune microenvironment can undergo dynamic changes under the selective pressure of EGFR‐TKI therapy [2, 7]. For example, studies have observed shifts in PD‐L1 expression and T‐cell infiltration between pre‐TKI biopsies and post‐TKI resistant tumors, reflecting an evolving immune landscape during treatment. In clinical practice, however, obtaining repeat tumor biopsies at the time of progression is often impractical because of inaccessible tumor sites or potential patient morbidity. This challenge highlights the need for alternative, noninvasive approaches, such as blood‐based profiling, to capture relevant biomarker information at the time of TKI resistance.

Peripheral blood provides a convenient, minimally invasive source for tumor biomarker discovery, and there is growing interest in the use of blood‐based multi‐omics analyses to explore tumor immune status. In particular, plasma proteomic profiling has become increasingly used to characterize systemic immune factors and identify predictors of immunotherapy response. For example, Christopoulos et al. showed that high‐throughput plasma proteomic profiling at baseline can stratify patients with advanced NSCLC into groups with high versus low probability of durable benefit from programmed cell death 1 (PD‐1)/PD‐L1 inhibitor‐based therapy [8]. Their multi‐protein classifier outperformed tumor PD‐L1 expression as a predictor of immunotherapy response. Additionally, elevated blood levels of inflammatory cytokines, such as interleukin (IL)‐6 and hepatocyte growth factor, have been associated with resistance to ICIs and poorer survival in patients with melanoma [9]. These findings underscore the potential of blood‐based proteomic biomarkers to noninvasively mirror the tumor immune microenvironment and improve patient selection for ICIs.

To address this unmet need, we conducted the present study to identify plasma‐based protein biomarkers at the time of EGFR‐TKI resistance that may predict response to subsequent ICI therapy in patients with EGFR‐mutant NSCLC.

## 2 Methods

### 2.1 Study Population and Response Classification

**FIGURE 1:**
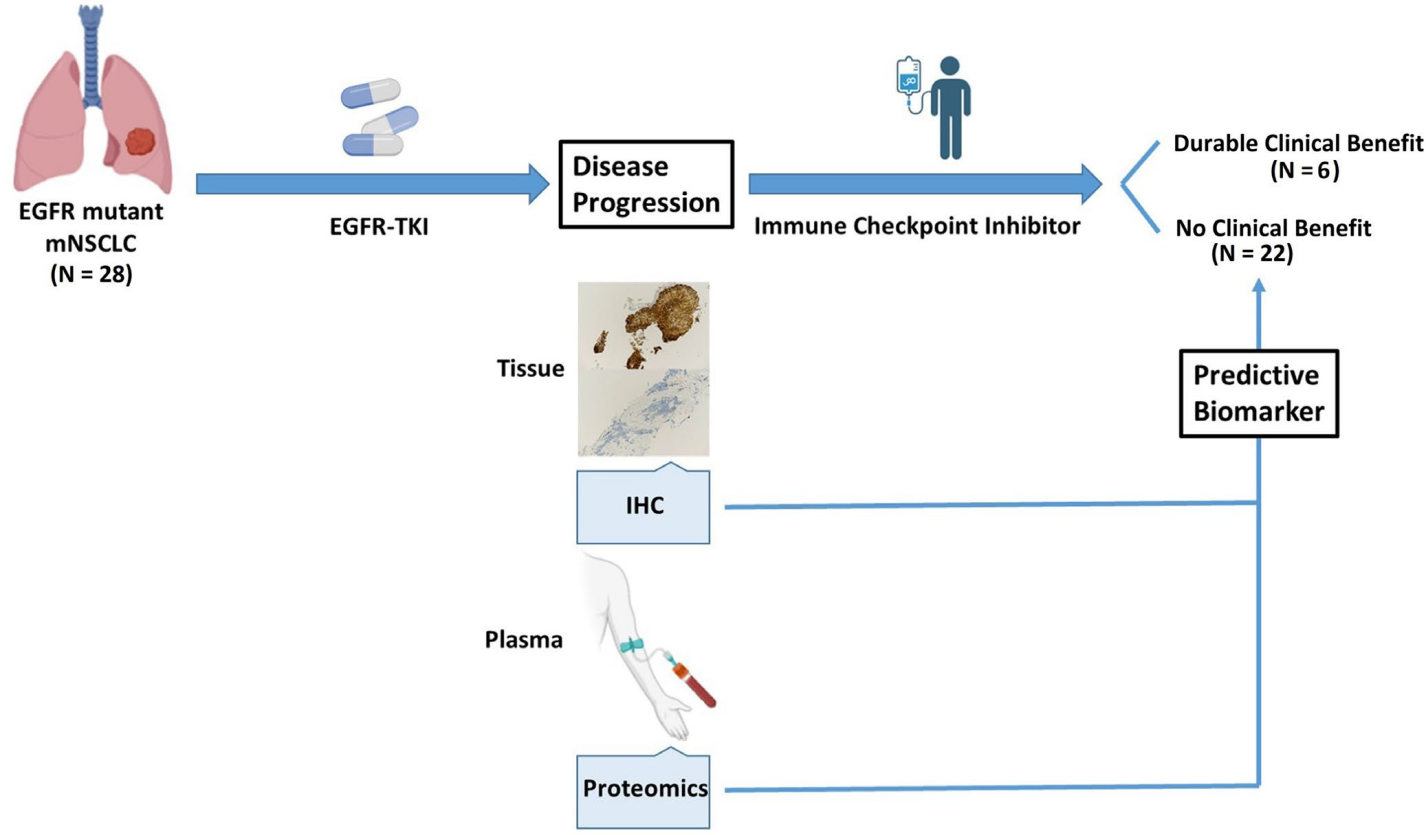
Overall schematic of the study workflow. Plasma samples were collected at the time of EGFR‐TKI progression, prior to ICI initiation.

We conducted a retrospective study of 28 patients with advanced EGFR‐mutant NSCLC who were treated at Inha University Hospital between November 2013 and September 2024 and received an ICI after developing resistance to EGFR‐TKI therapy (Figure 1). All patients had a confirmed activating EGFR mutation and experienced disease progression on first‐line or subsequent EGFR‐TKI therapy. After TKI failure, each patient received an anti‐PD‐1 or anti‐PD‐L1 ICI as the next line of therapy.

In this study, treatment benefit was assessed using durable clinical benefit (DCB) rather than RECIST‐based best overall response. Patients were categorized as having DCB if they achieved a progression‐free survival (PFS) of ≥ 6 months during ICI therapy, whereas those with PFS < 6 months were classified as having no clinical benefit (NCB). PFS was calculated from the date of the first ICI dose until radiographic or clinical disease progression or death. Patients alive without progression were censored at their last follow‐up visit. Baseline clinical data (age, sex, smoking history, histology, EGFR mutation subtype, PD‐L1 expression, and treatment) were collected for all patients. The protocol for this study was approved by the Institutional Review Board (IRB) committee of Inha University Hospital (IRB No. 2023‐03‐032), and informed consent was obtained from all patients.

### 2.2 Plasma Sample Collection and Proteomic Profiling

Peripheral blood samples were obtained from each patient at the time of acquired resistance to EGFR‐TKI therapy, prior to initiating ICI treatment. Blood was collected in ethylenediaminetetraacetic acid tubes and processed within 1 h to isolate plasma, which was aliquoted and stored at −80°C until analysis. Proteomic profiling of plasma was performed using the Olink Target 96 Immuno‐Oncology panel. All samples were run in one batch according to the manufacturer's protocol to minimize inter‐assay variability. The results were expressed as normalized protein expression (NPX) values, which are log base‐2 transformed. Internal and external controls included in the Olink panel were used to assess data quality, and proteins with NPX values below the limit of detection in > 20% of samples were excluded from analysis. Differential expression analysis was then performed to compare plasma protein levels between DCB and NCB. A volcano plot was generated to visualize the magnitude and significance of differences in protein levels between DCB and NCB. Proteins meeting predefined significance criteria (*p* < 0.1) were considered candidates for further validation.

Given the limited sample size, plasma proteomic analyses were conducted in an exploratory, hypothesis‐generating manner. Nominal *p*‐values were used to identify signals of potential interest, and no formal multiple‐testing correction was applied.

Candidate proteins were prioritized using an exploratory, multidimensional selection framework, which incorporated nominal statistical differences between the DCB and NCB groups, biological plausibility based on prior immunological literature, concordant expression patterns observed in both plasma and post EGFR‐TKI tumor tissue, and context specificity at the time of EGFR‐TKI resistance. This approach was intended to identify candidate biomarkers for subsequent validation rather than to establish definitive statistical associations.

### 2.3 Immunohistochemistry of Tumor Tissue

Formalin‐fixed paraffin‐embedded tumor tissue samples obtained after the development of EGFR‐TKI resistance were available for a subset of patients and were used for immunohistochemistry (IHC) analysis of the four candidate biomarkers. Tissue sections (4 μm) were cut from representative tumor blocks and mounted on charged slides. After deparaffinization and dehydration, antigen retrieval was performed by heating sections in citrate buffer (pH 6.0) for 20 min. The slides were then incubated with primary antibodies specific for galectin‐9 (Gal‐9), granzyme H (GZMH), IL‐4, and IL‐6 (1:50 dilution) for 1 h at room temperature. Horseradish peroxidase‐conjugated secondary antibody (1:100 dilution) and 3,3′‐diaminobenzidine chromogen were used for visualization of antibody binding, followed by counterstaining with hematoxylin. Negative control slides (omitting the primary antibody) were included in each run to confirm staining specificity.

Expression of each marker was evaluated independently by a board‐certified pathologist who was blinded to the clinical outcomes. Staining intensity and proportion of positively stained cells were evaluated, and IHC scores were assigned as follows: score 1, weak staining in < 50% of cells or moderate staining in < 20% of cells; score 2, weak staining in ≥ 50% of cells, moderate staining in 20%–50% of cells, or strong staining in < 20% of cells; or score 3, moderate staining in ≥ 50% of cells or strong staining in ≥ 20% of cells. This scoring system is a composite assessment of staining intensity and the percentage of positive cells in the tumor tissue. For each marker, tumors with an IHC score ≥ the median value for the cohort were classified as “high” expression, whereas tumors with a score below the median were categorized as “low” expression.

Plasma IL‐6 levels were interpreted as reflecting systemic inflammatory and immune status, whereas IL‐6 immunohistochemistry was used to assess local cytokine expression within the tumor microenvironment, including tumor and stromal cells. For IL‐6 immunohistochemistry, a rabbit polyclonal anti‐IL‐6 antibody (Abcam, ab6672) was used at a dilution of 1:30. Antigen retrieval conditions and antibody dilution were optimized empirically, and tissues with inflammatory features known to express IL‐6 were used as an internal reference to assess staining specificity. Antibody specificity was further confirmed in a lung cancer xenograft model (A549), which is known to express IL‐6. Negative controls were prepared by omitting the primary antibody. Based on prior immunohistochemical literature, IL‐6 expression has been reported to show weak to moderate diffuse cytoplasmic staining rather than strong, sharply localized tumor‐specific signals [10].

Immunohistochemical evaluation was performed by a board‐certified pathologist (L.K.), who was blinded to all clinical information.

### 2.4 Statistical Analysis

Patient characteristics and clinical outcomes were compared between DCB and NCB using descriptive statistics. Categorical variables were analyzed with Fisher's exact test, and continuous variables were analyzed using the Mann–Whitney *U* test. The association between plasma protein levels and DCB/NCB status was evaluated by performing differential expression analysis, as described above. PFS was estimated using the Kaplan–Meier method, and survival curves were compared using the log‐rank test. For biomarker analyses, patients were stratified into high versus low groups based on the median plasma level or IHC score of each candidate protein. All statistical tests were two‐sided, and *p* values < 0.05 were considered statistically significant. Data analyses were performed using SPSS (version 23.0; SPSS Inc., Chicago, IL, USA).

## 3 Results

### 3.1 Patient Characteristics

**FIGURE 2:**
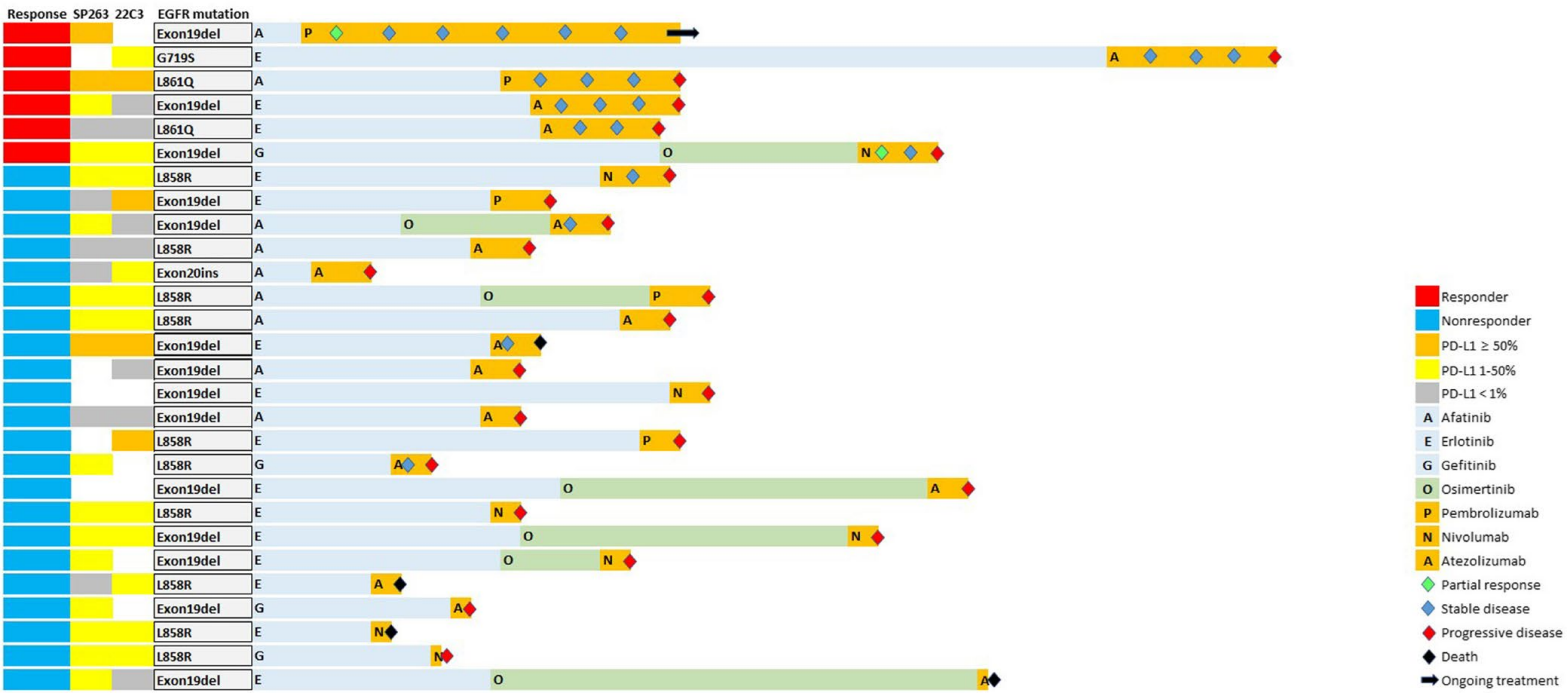
Clinical profiles of patients with EGFR‐TKI‐resistant NSCLC treated with an immune checkpoint inhibitor. Patients are ordered according to progression‐free survival following initiation of immune checkpoint inhibitor therapy.

Key baseline characteristics of the 28 patients included in this study are summarized in Table 1. Their median age was 59.5 years (interquartile range, 55.0–67.3 years), and 16 patients (57.1%) were male. The tumors of all patients contained an EGFR sensitizing mutation: 14 (50.0%) had an exon 19 deletion and 10 (35.7%) had an L858R point mutation. Before ICI therapy, all patients received a first‐ or second‐generation EGFR‐TKI (gefitinib, erlotinib, or afatinib), and 7 patients (25.0%) were subsequently treated with osimertinib (Figure 2). Following progression on a first‐ or second‐generation EGFR‐TKI, the T790M resistance mutation was detected in 11 patients (39.3%). After acquisition of EGFR‐TKI resistance, all patients received ICI monotherapy as the next line of therapy: 13 (46.4%) were treated with an anti‐PD‐1 antibody (pembrolizumab or nivolumab) and 15 (53.6%) received an anti‐PD‐L1 antibody (atezolizumab). ICIs were administered predominantly as later‐line therapy following EGFR‐TKI failure. Specifically, ICIs were given as third‐line treatment in 21 patients, fourth‐line in 4 patients, and fifth‐line or later in 3 patients. The median duration of prior EGFR‐TKI therapy was 13.2 months (range, 2.5–43.2 months). As summarized in Table 1, patients in this cohort exhibited heterogeneity with respect to EGFR‐TKI regimens, ICI agents, EGFR mutation subtypes, and PD‐L1 expression levels.

**TABLE 1:** Baseline patient and tumor characteristics.

Characteristic		No. of patients (%)
Age (year), median (IQR)		59.5 (55.0–67.3)
Sex	Male	16 (57.1)
	Female	12 (42.9)
Smoking history	Never	11 (39.3)
	Current or past	17 (60.7)
Histology	Adenocarcinoma	25 (89.3)
	Squamous cell carcinoma	1 (3.6)
	Adenosquamous carcinoma	1 (3.6)
	NSCLC‐NOS	1 (3.6)
*EGFR* mutation	Exon 19 del	14 (50.0)
	L858R	10 (35.7)
	Exon 20 ins	1 (3.6)
	L861Q	2 (7.1)
	G719S	1 (3.6)
T790M mutation	Acquired T790M	11 (39.3)
	Not detected	17 (60.7)
	De novo	0 (0.0)
PD‐L1 expression by SP263	≥ 50%	3 (10.7)
	1%–49%	15 (53.6)
	< 1%	5 (17.9)
	Not tested	5 (17.9)
PD‐L1 expression by 22C3	≥ 50%	7 (25.0)
	1%–49%	11 (39.3)
	< 1%	4 (14.3)
	Not tested	6 (21.4)

Abbreviations: EGFR, epidermal growth factor receptor; IQR, interquartile range; NSCLC‐NOS, non‐small cell lung cancer‐not otherwise specified.

Median PFS for the entire cohort was 2.4 months (95% confidence interval [CI], 1.5–3.3). Six patients (21.4%) were classified as DCB. Among these DCB, 2 achieved a partial response and 4 experienced durable stable disease. Of the 22 NCB, 15 exhibited progressive disease at the first assessment. Median PFS was 9.2 months for DCB (95% CI, 6.7–11.7 months) and 1.8 months for NCB (95% CI, 1.6–2.0 months) (*p* < 0.001). Most baseline characteristics (age, sex, smoking status, and PD‐L1 tumor expression) did not differ significantly between the two groups. However, uncommon EGFR mutations (L861Q, G719S, or exon 20 insertion) were significantly more frequent in NCB than in DCB.

### 3.2 Plasma Proteomic Differences Between DCB and NCB

**FIGURE 3:**
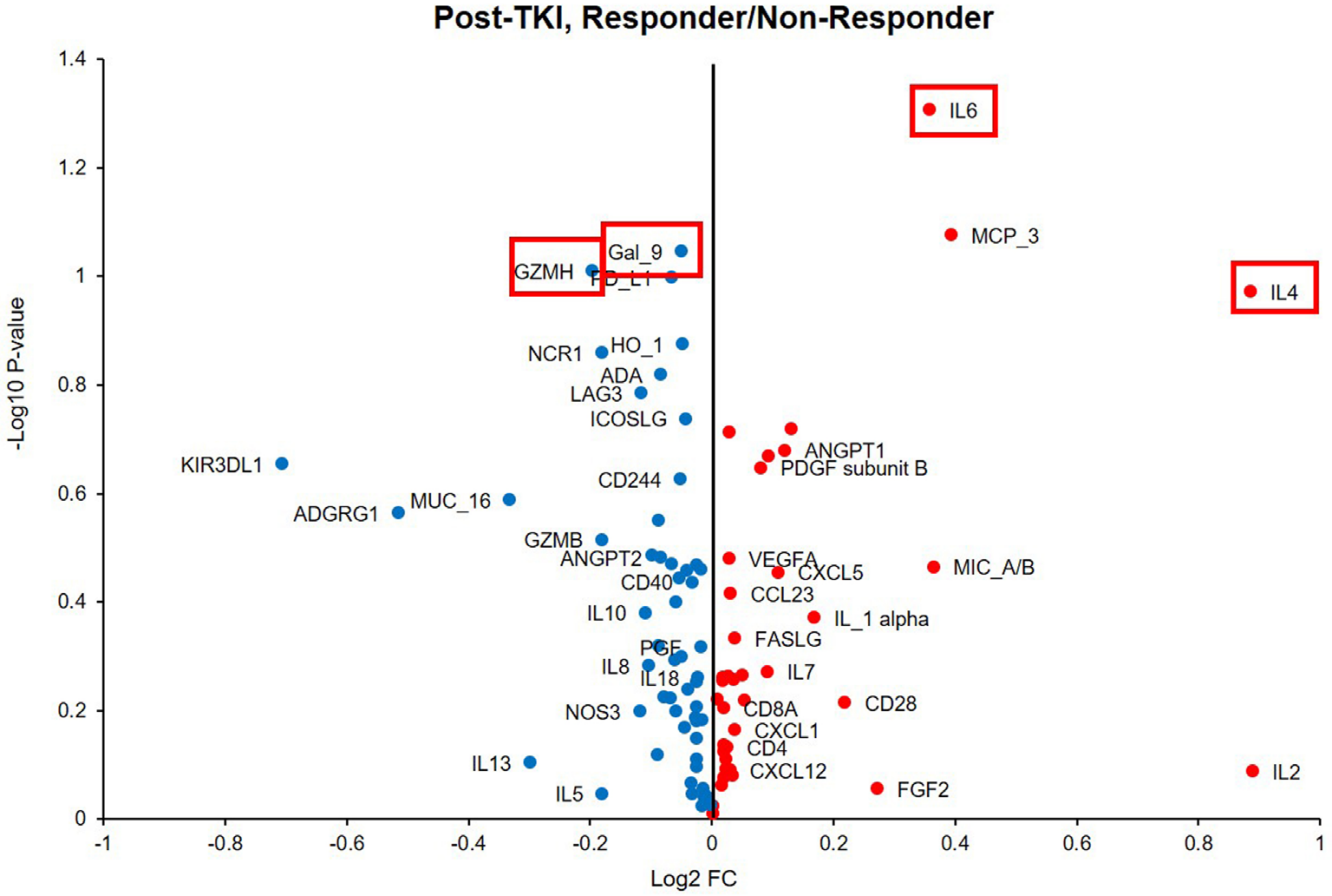
Volcano plot comparing post‐EGFR‐TKI plasma proteomic levels between durable clinical benefit (DCB) and no clinical benefit (NCB). Red dots indicate proteins upregulated in DCB, whereas blue dots indicate proteins upregulated in NCB. Nominal *p*‐values were used for exploratory analysis, and candidate proteins highlighted in boxes were prioritized based on a multidimensional selection framework incorporating statistical trends, biological plausibility, and consistency across plasma and tissue analyses, rather than a single predefined statistical threshold.

Exploratory proteomic analysis of plasma samples obtained at the time of EGFR‐TKI resistance identified several immune‐related proteins that showed differential expression patterns between the DCB and NCB groups among the 92 proteins tested. Based on nominal statistical differences and biological relevance, four proteins with potential association with ICI outcomes, namely Gal‐9, GZMH, IL‐4, and IL‐6, were prioritized for further analysis. A volcano plot summarizing these exploratory findings is shown in Figure 3. Proteins highlighted in the volcano plot were prioritized based on the exploratory, multidimensional selection framework described above rather than on predefined fold‐change or multiple‐testing adjusted significance thresholds.

**FIGURE 4:**
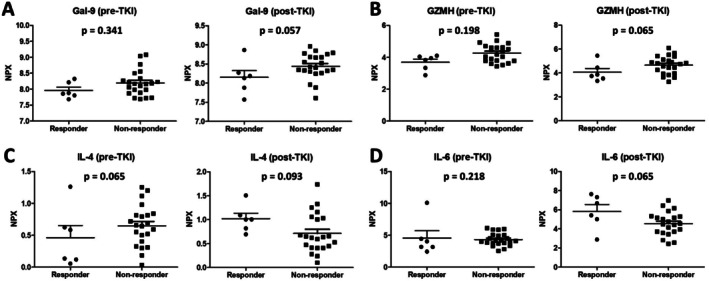
Comparison of proteomic levels for selected markers in pre‐ and post‐TKI plasma samples between durable clinical benefit (DCB) and no clinical benefit (NCB). (A) Gal‐9, (B) GZMH, (C) IL‐4, and (D) IL‐6. Gal‐9, galectin‐9; GZMH, granzyme H; IL, interleukin; TKI, tyrosine kinase inhibitor.

In quantitative analyses, IL‐4 and IL‐6 levels tended to be higher in the DCB group, whereas Gal‐9 and GZMH levels tended to be higher in the NCB group. By contrast, these four candidate proteins did not demonstrate differential expression between groups when assessed in pre‐TKI baseline plasma samples. These patterns are further illustrated in Figure 4, which shows the distribution of plasma expression levels of the four candidate markers in DCB versus NCB.

Patients with durable clinical benefit represented a heterogeneous clinical subset with respect to EGFR mutation subtype, EGFR‐TKI regimen, ICI agent, and PD‐L1 expression, precluding meaningful stratified statistical analyses.

### 3.3 Tumor Tissue Expression of Candidate Biomarkers

**FIGURE 5:**
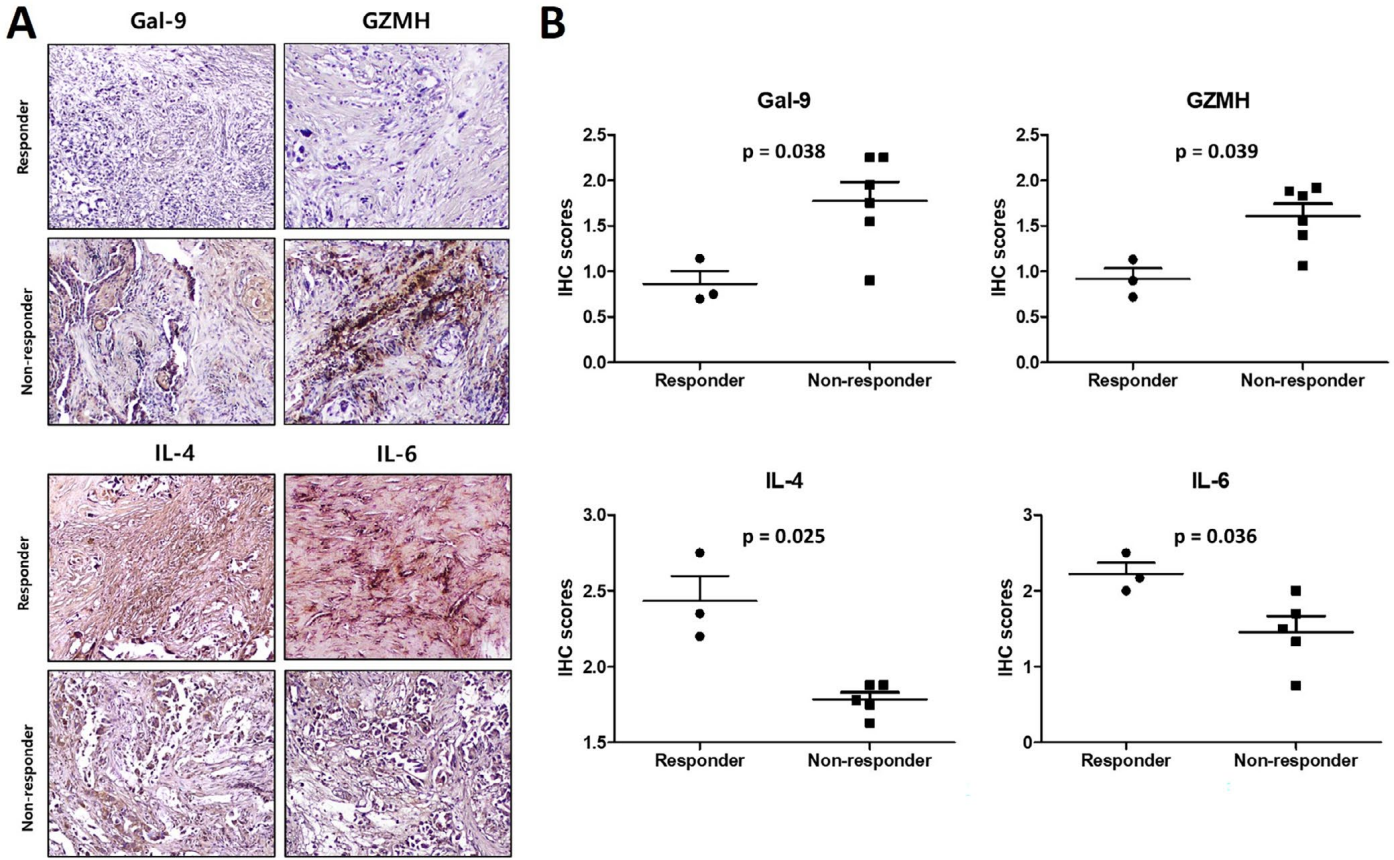
Immunohistochemical analysis of post‐EGFR‐TKI tissue samples. (A) Expression of Gal‐9, GZMH, IL‐4, and IL‐6 in durable clinical benefit (DCB) versus no clinical benefit (NCB). Representative immunohistochemical staining images of Gal‐9, GZMH, IL‐4, and IL‐6 in tumor samples (400×). (B) Scores based on the intensity and percentage of stained cells. Gal‐9, galectin‐9; GZMH, granzyme H; IL, interleukin.

To validate and further explore the identified potential biomarkers, we performed IHC analysis to examine the expression of Gal‐9, GZMH, IL‐4, and IL‐6 in tumor tissue obtained after the development of EGFR‐TKI resistance. Tumor samples from 9 patients (3 DCB and 6 NCB) were available for analysis. Representative immunostaining images for each marker are shown in Figure 5A. The distribution of IHC scores was generally consistent with the plasma results (Figure 5B). IHC scores for Gal‐9 and GZMH were significantly higher in the NCB group, whereas scores for IL‐4 and IL‐6 were significantly higher in the DCB group. Tissue immunohistochemistry was performed to provide contextual information regarding local expression patterns of the candidate proteins. These findings were not intended to indicate direct equivalence with circulating plasma levels, but rather to offer complementary insight into the tumor microenvironment. Given the secreted nature of IL‐6 and the potential for background staining, IL‐6 immunohistochemistry findings should be interpreted cautiously and were not intended to serve as definitive validation of plasma IL‐6 levels.

### 3.4 Association Between Biomarker Expression and Clinical Outcomes

**FIGURE 6:**
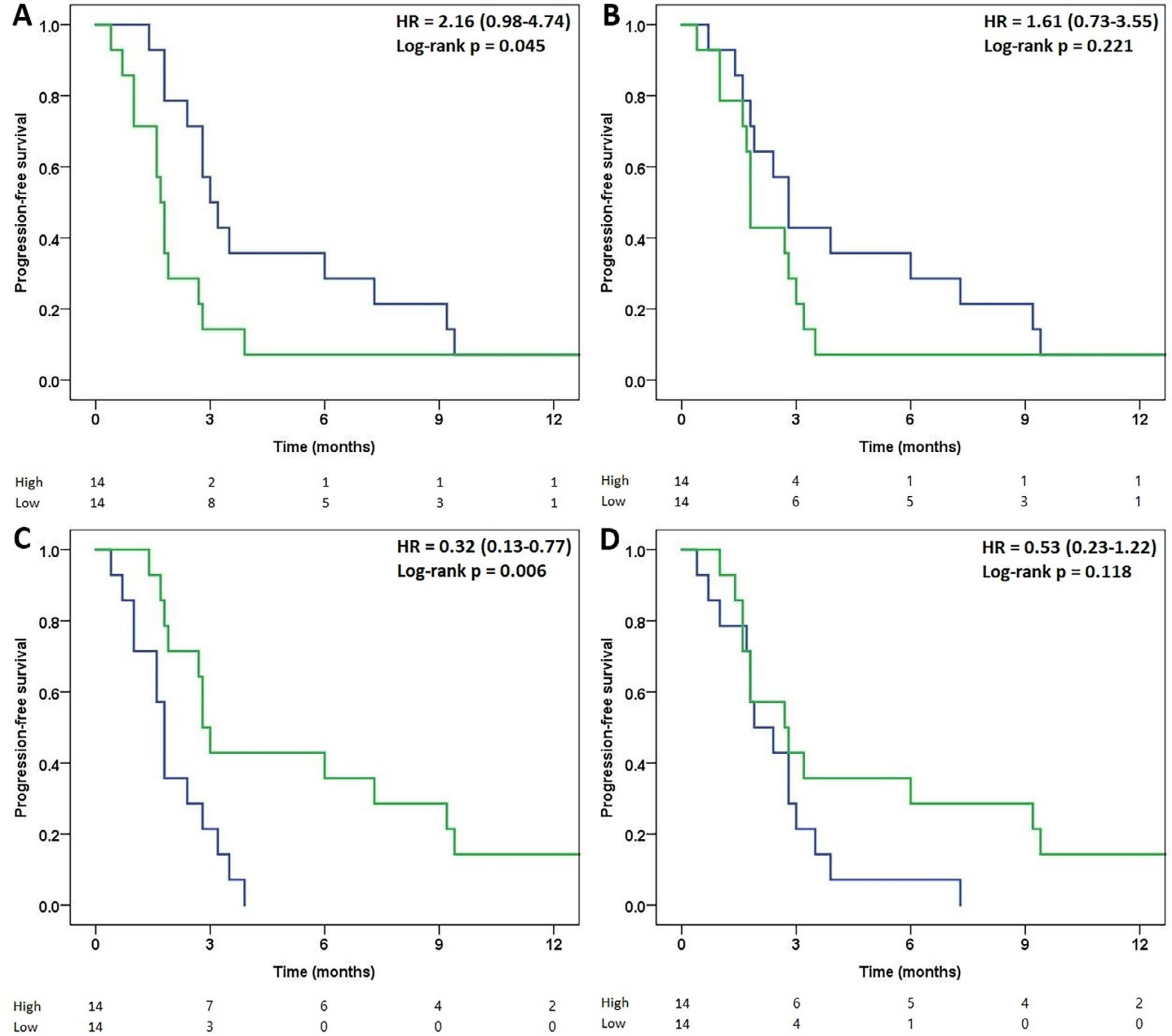
Comparison of progression‐free survival for high and low expression levels of (A) Gal‐9, (B) GZMH, (C) IL‐4, and (D) IL‐6. Green line, high expression (≥ median value); blue line, low expression (< median value). Hazard ratios with 95% confidence intervals were estimated using univariable Cox proportional hazards models, and log‐rank *p*‐values are shown. Numbers at risk are displayed below each curve. Gal‐9, galectin‐9; GZMH, granzyme H; IL, interleukin.

Kaplan–Meier analysis showed that the association between biomarker expression and PFS after ICI therapy differed between the four proteins (Figure 6). Patients with high Gal‐9 levels had a significantly shorter PFS than those with low Gal‐9 levels (median, 1.7 months [95% CI, 1.5–1.9 months] versus 3.0 months [95% CI, 2.7–3.7 months]; log‐rank *p* = 0.045). In univariable Cox proportional hazards analysis, high Gal‐9 expression was associated with an increased risk of progression. However, this association did not reach conventional statistical significance, with a wide confidence interval (hazard ratio = 2.16, 95% confidence interval = 0.98–4.74), reflecting limited statistical power. GZMH exhibited a similar trend, although the difference between expression groups did not reach statistical significance (1.8 months [95% CI, 1.6–1.9 months] versus 2.8 months [95% CI, 2.1–3.5 months]; log‐rank *p* = 0.221). Conversely, high IL‐4 expression was associated with longer PFS (2.8 months [95% CI, 2.4–3.2 months] versus 1.8 months [95% CI, 1.6–2.0 months]; log‐rank *p* = 0.006). The same directional trend was observed for high versus low IL‐6 expression, albeit without statistical significance (2.7 months [95% CI, 0.9–4.5 months] versus 1.9 months [95% CI, 0.8–3.0 months]; log‐rank *p* = 0.118).

## 4 Discussion

In this study, we identified several plasma protein biomarkers that distinguish patients with EGFR‐mutant NSCLC who benefit from ICI therapy after developing TKI resistance. Specifically, we found that Gal‐9, GZMH, IL‐4, and IL‐6 were differentially expressed between DCB and NCB at the time of development of EGFR‐TKI resistance, whereas no such differences were observed in pre‐TKI baseline samples. Importantly, treatment benefit in this study was evaluated using durable clinical benefit rather than RECIST‐based objective response. This approach was chosen because objective responses to ICIs are rare in EGFR‐mutant NSCLC after EGFR‐TKI resistance, and prolonged disease stabilization is more clinically meaningful in this setting. IHC analysis of post‐TKI tumor specimens corroborated these findings, confirming higher Gal‐9 and GZMH expression in NCB and greater IL‐4 and IL‐6 in DCB. Furthermore, lower Gal‐9 and higher IL‐4 levels at the time of TKI resistance were significantly associated with longer PFS during subsequent immunotherapy, highlighting their potential as predictive biomarkers of treatment response. Collectively, these findings suggest that the tumor immune microenvironment of EGFR‐mutant NSCLC undergoes notable changes upon the development of TKI resistance, and measuring key immunomodulatory proteins at this time can help identify patients most likely to respond to ICI therapy.

Our results address an important gap in the management of EGFR‐mutant lung cancer. Patients with EGFR‐mutant NSCLC have historically responded poorly to PD‐1/PD‐L1 inhibitors, likely secondary to their “cold” tumor immune profile [1]. Currently, there are no reliable biomarkers to guide immunotherapy use when TKIs fail in this subset of patients. Our study is one of the first to focus on this TKI‐refractory setting and to use unbiased proteomic profiling for biomarker discovery. The identification of Gal‐9, GZMH, IL‐4, and IL‐6 as response‐associated factors is novel and reveals that the selective pressure of EGFR‐TKI therapy can create immunologic divergence between patients that was not apparent at baseline. This dynamic insight—that predictive immune biomarkers may emerge over the course of targeted treatment—is a key contribution to the field. It suggests that timing and disease context are of critical importance when evaluating immunotherapy biomarkers in oncogene‐driven cancers.

Gal‐9 emerged as a compelling candidate biomarker, and its higher levels in nonresponders point to a biologically plausible mechanism of immune escape [11]. Gal‐9 is a lectin and known ligand for T‐cell immunoglobulin and mucin domain‐containing protein 3 (TIM‐3), a T‐cell inhibitory receptor. Gal‐9 binding to TIM‐3 induces apoptosis of effector T cells, thereby suppressing anti‐tumor immunity [12]. Tumors often exploit this pathway, and Gal‐9 is frequently upregulated in cancer cells, where it contributes to T‐cell exhaustion and immune evasion. Yang et al. reported that high Gal‐9 expression in the tumor microenvironment correlates with poor prognosis in multiple cancers [12]. Our finding that NCB had increased Gal‐9 aligns with these observations, and an immunosuppressive, Gal‐9‐rich milieu likely hinders the efficacy of PD‐1 inhibitors. Taken together, our results and those of prior studies support the biologic plausibility of Gal‐9 as a predictive biomarker of ICI resistance in EGFR‐mutant NSCLC. It may be worthwhile exploring therapies targeting the Gal‐9/TIM‐3 axis in this patient population, as blocking Gal‐9 can enhance T‐cell survival and has shown promise when combined with other immunomodulators.

Our findings are supported by several clinical studies reporting that elevated IL‐4 levels are associated with superior outcomes during immunotherapy. In their study of 26 patients with NSCLC, Boutsikou et al. found that responders to PD‐1 inhibitors exhibited higher baseline levels of several cytokines, including IL‐4, and that these elevations were associated with longer overall survival [13]. Similarly, a recent study by Su et al. reported that pretreatment serum IL‐4 was significantly higher in responders than in progressors, and patients with high IL‐4 levels experienced superior PFS during anti‐PD‐1 therapy [14].

These clinical correlations suggest that IL‐4 may mark an immune‐active state conducive to immunotherapy, despite its “Th2” label. Mechanistically, IL‐4 may contribute to anti‐tumor immunity in specific contexts. Ruggiu et al. demonstrated a novel role for IL‐4 in anti‐PD‐1 therapy: in a mouse model, PD‐1 inhibitor induced a surge of IL‐4 from T follicular helper cells in tumor‐draining lymph nodes, and this surge was required for optimal CD8^+^ T‐cell proliferation and function during therapy [15]. Blocking IL‐4 in these experiments abrogated the CD8^+^ T‐cell expansion normally driven by PD‐1 antibodies, whereas providing exogenous IL‐4 could rescue and mimic the pro‐immunogenic effects of PD‐1 inhibitors. These findings align with our observation that patients with higher IL‐4 levels responded better to ICI therapy than those with lower IL‐4 levels, suggesting that IL‐4 may act as a supportive cytokine for T‐cell priming or survival during checkpoint inhibition. The higher IL‐4 levels observed in DCB may reflect greater activity of IL‐4‐producing immune cells (e.g., T helper 2 cells, certain dendritic cell subsets) that help orchestrate an effective anti‐tumor response. Alternatively, the higher IL‐4 levels could represent a more general state of immune responsiveness or lymph node activation in these patients. In summary, while IL‐4 is traditionally associated with humoral immunity and tissue repair, emerging evidence suggests it plays a facilitative role in anti‐PD‐1 immune responses in vivo. Our data add clinical relevance to this concept, identifying IL‐4 as a potential biomarker of a tumor microenvironment that is permissive to ICI therapy, even in EGFR‐mutant NSCLC.

Our finding that GZMH expression was elevated in patients with EGFR‐mutant NSCLC who failed to respond to PD‐1/PD‐L1 inhibitor therapy is somewhat unexpected, based on the results of previous studies. GZMH is a cytotoxic serine protease predominantly expressed by natural killer (NK) cells and cytotoxic T lymphocytes. It is capable of inducing caspase‐dependent apoptosis in target cells [16], and robust GZMH expression is usually a hallmark of active anti‐tumor immunity. For example, loss‐of‐function alterations in granzyme genes have been linked to poor immunotherapy outcomes. In a cohort of patients with nasopharyngeal carcinoma treated with anti‐PD‐1, individuals with *GZMH* gene deletions had significantly shorter PFS and overall survival on ICI therapy [17]. Similarly, low intratumoral GZMH expression was associated with worse prognosis, and high combined granzyme family expression correlated with better survival in a cohort of patients with melanoma [18]. These observations suggest that higher GZMH levels typically reflect a more effective cytotoxic immune infiltrate, which is usually associated with a better response to ICI therapy.

Thus, the higher GZMH expression observed in NCB contrasts with the prevailing trend seen in other settings. One possible explanation is that GZMH may reflect an inefficient or dysregulated immune response in EGFR‐driven tumors. EGFR‐mutant NSCLCs often exhibit an immune milieu skewed away from productive T‐cell immunity, such as low MHC‐I expression and high levels of immunosuppressive factors, which favor tumor evasion. In this context, an abundance of GZMH‐positive cytotoxic cells may represent a compensatory innate response that is inadequate to produce tumor regression. Indeed, NK cells can infiltrate EGFR‐mutant tumors when adaptive responses are blunted, but these NK cells may become functionally exhausted or inhibited in the tumor microenvironment, rendering their GZMH release ineffective [19]. That is, elevated GZMH levels in NCB may be a sign of an attempted immune attack that fails, perhaps reflecting high intratumoral NK/T‐cell activity that is being counteracted by tumor‐associated inhibitory signals. Increased GZMH expression may reflect dysfunctional cytotoxicity or compensatory NK/T‐cell infiltration in immunosuppressive tumors. This interpretation aligns with the concept that prominent immune cell infiltration is not necessarily synonymous with effective anti‐tumor immunity in EGFR‐mutant NSCLC but may instead reflect an ineffective or exhausted immune response. Thus, while the literature supports high granzyme expression as generally beneficial for immunotherapy, our findings suggest that in EGFR‐mutant NSCLC, the location, source, and context of GZMH expression determine whether it translates into effective tumor immunity.

Importantly, circulating IL‐6 levels measured in plasma and IL‐6 expression assessed by tissue immunohistochemistry represent biologically distinct measurements. Plasma IL‐6 primarily reflects systemic inflammatory or immune activity, whereas tissue IL‐6 immunohistochemistry captures local cytokine production within the tumor microenvironment. These two assessments should not be interpreted as representing the same biological process. Our study also revealed that patients with EGFR‐mutant NSCLC who responded to ICIs exhibited elevated plasma and tissue levels of IL‐6 at the time of developing EGFR‐TKI resistance. This observation appears paradoxical, as IL‐6 is widely regarded as a pro‐inflammatory cytokine implicated in tumor progression, immune evasion, and poor prognosis in various cancers, including NSCLC [9, 20]. Elevated IL‐6 levels are commonly associated with resistance to immunotherapy. For example, Kang et al. found that high baseline IL‐6 levels were associated with significantly lower response rates and shorter survival during anti‐PD‐1 therapy in patients with NSCLC [21]. IL‐6 can activate signal transducer and activator of transcription 3 signaling in the tumor microenvironment, driving immunosuppressive effects, such as impaired dendritic cell antigen presentation and blunted Th1 T‐cell responses. Preclinical studies demonstrated that IL‐6 fosters ICI resistance in various models and combining an IL‐6 inhibitor with PD‐1/PD‐L1 inhibitors enhances anti‐tumor efficacy. In addition, interpretation of tissue IL‐6 immunohistochemistry is inherently limited by the secreted nature of IL‐6, low intracellular retention, and the possibility of nonspecific background staining, and these technical considerations further support a cautious interpretation of tissue‐based findings.

Recent literature has strongly supported IL‐6 as a mediator of immunotherapy resistance, particularly through activation of the IL‐6/IL‐6R/gp130‐JAK/STAT3 signaling axis, which promotes the development of an immunosuppressive tumor microenvironment. This signaling cascade has been implicated in multiple mechanisms that impair effective antitumor immunity, including suppression of antigen presentation and attenuation of cytotoxic T‐cell‐mediated immune responses [22, 23]. In parallel, IL‐6 is a central cytokine in the biology of cancer cachexia, and cachexia has consistently been associated with inferior clinical outcomes in patients treated with ICIs, including those with NSCLC [24, 25]. Therefore, our observation that higher post‐TKI IL‐6 levels were enriched in the DCB group should be interpreted with caution and explicitly contrasted with this prevailing paradigm. In addition, IL‐6 signaling requires engagement of the IL‐6 receptor and downstream activation of the gp130‐STAT3 pathway. Therefore, measurement of the IL‐6 ligand alone may not fully capture pathway activity within the tumor microenvironment. Assessment of IL‐6 receptor expression or downstream signaling markers, such as phosphorylated STAT3, may provide more biologically informative insight and should be considered in future studies.

One possible reconciliation is that the clinical significance of circulating IL‐6 depends on timing and host context. In the present study, plasma samples were obtained at the time of EGFR‐TKI progression immediately prior to ICI initiation, rather than in an ICI‐naïve setting. In this post‐TKI context, elevated IL‐6 levels may reflect a systemic immune‐reactive inflammatory state associated with tumor evolution or tissue injury, which may coexist with effective immune priming during subsequent checkpoint blockade, rather than chronic tumor‐driven IL‐6 signaling that sustains immune escape [22, 26]. Importantly, we do not interpret IL‐6 as a direct mechanistic driver of immunotherapy sensitivity. Instead, IL‐6 is proposed as an exploratory, context‐dependent surrogate marker that may capture host inflammatory or immune readiness in a subset of patients with EGFR‐mutant NSCLC after the development of TKI resistance.

However, evidence also exists indicating that the role of IL‐6 in immunity is context‐dependent. Notably, a clinical study in advanced melanoma found that pretreatment IL‐6 and interferon‐γ levels were higher in patients who responded to nivolumab than in individuals who were nonresponders [26]. In a small cohort of patients with NSCLC, anti‐PD‐1 responders had higher baseline IL‐6 levels than nonresponders [13], and in a recent analysis of 88 patients with NSCLC, high IL‐6 levels at baseline were associated with significantly longer PFS during anti‐PD‐1 therapy [14]. These results, therefore, support IL‐6 as a marker of ICI treatment response and improved outcomes in certain settings, despite its traditional reputation. Taken together, it appears that IL‐6 can function at both ends of the spectrum: while chronic tumor‐derived IL‐6 signaling promotes immune evasion, an acute increase in IL‐6 during immune activation may simply reflect vigorous inflammation accompanying tumor control. IL‐6 is integral to initiating immune responses, as it helps drive T‐cell proliferation and differentiation. Therefore, the elevated IL‐6 levels observed in DCB may be a byproduct of a highly inflamed, immune‐reactive tumor microenvironment. Elevated IL‐6 levels in DCB may reflect acute‐phase immune activation associated with vigorous T‐cell priming and proliferation during effective immune responses. In this context, IL‐6 may act as a marker of immune system engagement, rather than a driver of immune evasion.

Despite our encouraging findings, this study has limitations. First, this was a retrospective, single‐center analysis with a modest sample size, and all patients were derived from a single‐center cohort. The inherently small number of DCB in the EGFR‐mutant, post‐TKI setting limits the statistical power of our analyses. In addition, the evaluation of a large number of plasma proteins without formal multiple‐testing correction increases the risk of false‐positive findings. Therefore, our findings should be regarded as exploratory and hypothesis‐generating, and external validation in independent, prospective cohorts is required before any clinical application. Larger multicenter studies are necessary to confirm Gal‐9, GZMH, IL‐4, and IL‐6 as predictive biomarkers. Second, we did not stratify the tumors according to TKI resistance mechanisms. EGFR‐mutant tumors can acquire TKI resistance through diverse biologic processes, which may differentially reshape the tumor immune microenvironment. Thus, the predictive value of our plasma biomarkers may vary across different subgroups. For instance, patients with small‐cell transformation may display a profoundly different cytokine profile than patients with persistent T790M‐mediated resistance. Future studies should evaluate whether the identified biomarkers retain prognostic relevance within distinct resistance mechanism strata. In addition, clinical heterogeneity with respect to EGFR‐TKI regimens, ICI agents, EGFR mutation subtypes, and PD‐L1 expression may have confounded associations between plasma cytokine levels and clinical outcomes. Although comprehensive multivariable adjustment was not feasible due to sample size constraints, this limitation further underscores the exploratory nature of our findings. Third, we can only report associations, not causal relationships, given the observational design of our study. Although we interpreted higher IL‐4 and lower Gal‐9 levels as potentially facilitating better response to ICI therapy, it remains possible that these protein changes are simply downstream effects, reflecting effective immune activation rather than directly contributing to it. We attempted to strengthen the evidence by performing tissue IHC analysis, but functional experiments were beyond the scope of this study. Thus, the mechanistic roles of the identified biomarkers remain to be validated experimentally. Fourth, we lacked systematic cachexia‐ and inflammation‐related clinical covariates, which limits our ability to distinguish chronic cachexia‐associated IL‐6 elevation from immune‐reactive IL‐6 signaling. Given the established association between IL‐6, cancer cachexia, and resistance to immune checkpoint inhibition, our findings regarding IL‐6 should be considered hypothesis‐generating and require external validation in independent cohorts incorporating integrated clinical and mechanistic correlates. Furthermore, methodological limitations inherent to IL‐6 immunohistochemistry restrict definitive interpretation of tissue IL‐6 expression, and additional validation using alternative approaches would be required to assess IL‐6 signaling activity in the tumor microenvironment. Fifth, plasma proteomic measurements can be influenced by systemic factors unrelated to the tumor itself, such as infections or comorbid inflammatory conditions. While we mitigated potential confounding by analyzing matched tumor tissues and standardizing the timing of plasma collection, we cannot completely exclude systemic influences on biomarker levels. Finally, our focus on EGFR‐mutant NSCLC, while clinically important, means that our findings may not generalize to broader NSCLC populations or other oncogene‐driven cancers. This specificity is both a strength and a limitation, as it addresses a niche need but calls for cautious interpretation if extrapolated to other settings.

This study accomplished the objective of identifying a novel set of plasma biomarkers that may predict response to ICI following TKI resistance in patients with EGFR‐mutant NSCLC. The main implication is that even tumors traditionally considered less immunogenic can harbor or develop immunologic differences that impact immunotherapy outcomes. Measuring circulating protein levels at the time of TKI failure may help oncologists personalize subsequent treatment. A possible future direction of research would involve validating these biomarkers prospectively and optimizing predictive accuracy by generating a composite score or algorithm that integrates the biomarkers with other factors.

In summary, our study contributes to the evolving understanding that the immune landscape in oncogene‐driven NSCLC is not static and can be leveraged for guiding therapy. By identifying immune biomarkers unique to the post‐TKI resistant state, our results are a step toward more rational and tailored use of immunotherapy in EGFR‐mutant lung cancer, with the ultimate goal of improving outcomes in this challenging clinical setting.

## Author Contributions

**Min Seok Park:** conceptualization, data curation, formal analysis, investigation, methodology, writing – original draft, writing – review and editing. **Jun Hyeok Lim:** conceptualization, data curation, project administration, formal analysis, investigation, methodology, visualization, validation, writing – original draft, writing – review and editing. **Nuri Park:** data curation, investigation, validation, writing – review and editing. **Eunji Park:** data curation, investigation, validation, writing – review and editing. **Ayoung Lim:** data curation, investigation, validation, writing – review and editing. **Suji Lee:** data curation, investigation, validation, writing – review and editing. **Yejin Cho:** data curation, investigation, validation, writing – review and editing. **Sehan Kwak:** data curation, investigation, validation, writing – review and editing. **Minseo Lee:** data curation, investigation, validation, writing – review and editing. **Donghyun Seo:** data curation, investigation, validation, writing – review and editing. **Lucia Kim:** data curation, methodology, validation, writing – review and editing. **Woo Kyung Ryu:** data curation, methodology, validation, writing – review and editing. **Jeong‐Seon Ryu:** data curation, methodology, validation, writing – review and editing. **Eun Young Kim:** data curation, methodology, validation, writing – review and editing. **Soon‐Sun Hong:** data curation, methodology, validation, writing – review and editing. **Kyung Hee Jung:** conceptualization, data curation, formal analysis, investigation, methodology, visualization, validation, writing – original draft, writing – review and editing.

## Funding

This work was supported by Korean Association for the Study of Targeted Therapy (KASTT‐20220111), National Research Foundation of Korea (NRF) (RS‐2022‐NR071926), and Inha University.

## Conflicts of Interest

The authors declare no conflicts of interest.

## Data Availability

The data that support the findings of this study are available from the corresponding author upon reasonable request.

## References

[1] M. Qiao , T. Jiang , X. Liu , et al., “Immune Checkpoint Inhibitors in EGFR‐Mutated NSCLC: Dusk or Dawn?,” Journal of Thoracic Oncology 16, no. 8 (2021): 1267–1288. 33915248 10.1016/j.jtho.2021.04.003

[2] J. F. Gainor , A. T. Shaw , L. V. Sequist , et al., “EGFR Mutations and ALK Rearrangements Are Associated With Low Response Rates to PD‐1 Pathway Blockade in Non‐Small Cell Lung Cancer: A Retrospective Analysis,” Clinical Cancer Research 22, no. 18 (2016): 4585–4593. 27225694 10.1158/1078-0432.CCR-15-3101 PMC5026567

[3] X. Le , M. V. Negrao , A. Reuben , et al., “Characterization of the Immune Landscape of EGFR‐Mutant NSCLC Identifies CD73/Adenosine Pathway as a Potential Therapeutic Target,” Journal of Thoracic Oncology 16, no. 4 (2021): 583–600. 33388477 10.1016/j.jtho.2020.12.010 PMC11160459

[4] J. C.‐H. Yang , D. H. Lee , J.‐S. Lee , et al., “Phase III KEYNOTE‐789 Study of Pemetrexed and Platinum With or Without Pembrolizumab for Tyrosine Kinase Inhibitor‐Resistant, EGFR‐Mutant, Metastatic Nonsquamous Non‐Small Cell Lung Cancer,” Journal of Clinical Oncology 42, no. 34 (2024): 4029–4039. 39173098 10.1200/JCO.23.02747 PMC11608596

[5] T. Mok , K. Nakagawa , K. Park , et al., “Nivolumab Plus Chemotherapy in Epidermal Growth Factor Receptor‐Mutated Metastatic Non‐Small‐Cell Lung Cancer After Disease Progression on Epidermal Growth Factor Receptor Tyrosine Kinase Inhibitors: Final Results of CheckMate 722,” Journal of Clinical Oncology 42, no. 11 (2024): 1252–1264. 38252907 10.1200/JCO.23.01017 PMC11095864

[6] K. Hastings , H. Yu , W. Wei , et al., “EGFR Mutation Subtypes and Response to Immune Checkpoint Blockade Treatment in Non‐Small‐Cell Lung Cancer,” Annals of Oncology 30, no. 8 (2019): 1311–1320. 31086949 10.1093/annonc/mdz141 PMC6683857

[7] K. Isomoto , K. Haratani , H. Hayashi , et al., “Impact of EGFR‐TKI Treatment on the Tumor Immune Microenvironment in EGFR Mutation‐Positive Non‐Small Cell Lung Cancer,” Clinical Cancer Research 26, no. 8 (2020): 2037–2046. 31937613 10.1158/1078-0432.CCR-19-2027

[8] P. Christopoulos , M. Harel , K. McGregor , et al., “Plasma Proteome‐Based Test for First‐Line Treatment Selection in Metastatic Non‐Small Cell Lung Cancer,” JCO Precision Oncology 8 (2024): e2300555. 38513170 10.1200/PO.23.00555 PMC10965206

[9] N. Rossi , K. A. Lee , M. V. Bermudez , et al., “Circulating Inflammatory Proteins Associate With Response to Immune Checkpoint Inhibition Therapy in Patients With Advanced Melanoma,” eBioMedicine 83 (2022): 104235. 36007304 10.1016/j.ebiom.2022.104235 PMC9421308

[10] J. Zeng , Z.‐H. Tang , S. Liu , and S.‐S. Guo , “Clinicopathological Significance of Overexpression of Interleukin‐6 in Colorectal Cancer,” World Journal of Gastroenterology 23, no. 10 (2017): 1780–1786. 28348483 10.3748/wjg.v23.i10.1780 PMC5352918

[11] Y. Lv , X. Ma , Y. Ma , Y. Du , and J. Feng , “A New Emerging Target in Cancer Immunotherapy: Galectin‐9 (LGALS9),” Genes & Diseases 10, no. 6 (2023): 2366–2382. 37554219 10.1016/j.gendis.2022.05.020 PMC10404877

[12] R. Yang , L. Sun , C.‐F. Li , et al., “Galectin‐9 Interacts With PD‐1 and TIM‐3 to Regulate T Cell Death and Is a Target for Cancer Immunotherapy,” Nature Communications 12, no. 1 (2021): 832. 10.1038/s41467-021-21099-2 PMC7864927 33547304

[13] E. Boutsikou , K. Domvri , G. Hardavella , D. Tsiouda , K. Zarogoulidis , and T. Kontakiotis , “Tumour Necrosis Factor, Interferon‐Gamma and Interleukins as Predictive Markers of Antiprogrammed Cell‐Death Protein‐1 Treatment in Advanced Non‐Small Cell Lung Cancer: A Pragmatic Approach in Clinical Practice,” Therapeutic Advances in Medical Oncology 10 (2018): 1758835918768238. 29662549 10.1177/1758835918768238 PMC5894896

[14] S. Su , F. Chen , X. Lv , et al., “Predictive Value of Peripheral Blood Biomarkers in Patients With Non‐Small‐Cell Lung Cancer Responding to Anti‐PD‐1‐Based Treatment,” Cancer Immunology, Immunotherapy 73, no. 1 (2024): 12. 38231411 10.1007/s00262-023-03620-2 PMC10794255

[15] M. Ruggiu , M. V. Guérin , B. Corre , et al., “Anti‐PD‐1 Therapy Triggers Tfh Cell–Dependent IL‐4 Release to Boost CD8 T Cell Responses in Tumor‐Draining Lymph Nodes,” Journal of Experimental Medicine 221, no. 4 (2024): e20232104. 38417020 10.1084/jem.20232104 PMC10901238

[16] Q. Hou , T. Zhao , H. Zhang , et al., “Granzyme H Induces Apoptosis of Target Tumor Cells Characterized by DNA Fragmentation and Bid‐Dependent Mitochondrial Damage,” Molecular Immunology 45, no. 4 (2008): 1044–1055. 17765974 10.1016/j.molimm.2007.07.032

[17] Y. Ma , X. Chen , A. Wang , et al., “Copy Number Loss in Granzyme Genes Confers Resistance to Immune Checkpoint Inhibitor in Nasopharyngeal Carcinoma,” Journal for Immunotherapy of Cancer 9, no. 3 (2021): e002014. 33737344 10.1136/jitc-2020-002014 PMC7978327

[18] X. Wu , X. Wang , Y. Zhao , K. Li , B. Yu , and J. Zhang , “Granzyme Family Acts as a Predict Biomarker in Cutaneous Melanoma and Indicates More Benefit From Anti‐PD‐1 Immunotherapy,” International Journal of Medical Sciences 18, no. 7 (2021): 1657–1669. 33746582 10.7150/ijms.54747 PMC7976569

[19] H. Jia , H. Yang , H. Xiong , and K. Q. Luo , “NK Cell Exhaustion in the Tumor Microenvironment,” Frontiers in Immunology 14 (2023): 1303605. 38022646 10.3389/fimmu.2023.1303605 PMC10653587

[20] W. Ke , L. Zhang , and Y. Dai , “The Role of IL‐6 in Immunotherapy of Non‐Small Cell Lung Cancer (NSCLC) With Immune‐Related Adverse Events (irAEs),” Thoracic Cancer 11, no. 4 (2020): 835–839. 32043828 10.1111/1759-7714.13341 PMC7113041

[21] D. H. Kang , C.‐K. Park , C. Chung , et al., “Baseline Serum Interleukin‐6 Levels Predict the Response of Patients With Advanced Non‐Small Cell Lung Cancer to PD‐1/PD‐L1 Inhibitors,” Immune Network 20, no. 3 (2020): e27. 32655975 10.4110/in.2020.20.e27 PMC7327149

[22] M. F. Soler , A. Abaurrea , P. Azcoaga , A. M. Araujo , and M. M. Caffarel , “New Perspectives in Cancer Immunotherapy: Targeting IL‐6 Cytokine Family,” Journal for Immunotherapy of Cancer 11, no. 11 (2023): e007530. 37945321 10.1136/jitc-2023-007530 PMC10649711

[23] L. Paschold , C. Schultheiss , P. Schmidt‐Barbo , et al., “Inflammation and Limited Adaptive Immunity Predict Worse Outcomes on Immunotherapy in Head and Neck Cancer,” npj Precision Oncology 9, no. 1 (2025): 272. 40764401 10.1038/s41698-025-01020-6 PMC12325939

[24] N. Matsuo , K. Azuma , K. Murotani , et al., “Prognostic Effect of Cachexia in Patients With Non‐Small Cell Lung Cancer Receiving Immune Checkpoint Inhibitors,” Thoracic Cancer 14, no. 15 (2023): 1362–1367. 37037511 10.1111/1759-7714.14881 PMC10212657

[25] K. Rounis , D. Makrakis , A.‐P. Tsigkas , et al., “Cancer Cachexia Syndrome and Clinical Outcome in Patients With Metastatic Non‐Small Cell Lung Cancer Treated With PD‐1/PD‐L1 Inhibitors: Results From a Prospective, Observational Study,” Translational Lung Cancer Research 10, no. 8 (2021): 3538–3549. 34584855 10.21037/tlcr-21-460 PMC8435387

[26] N. Yamazaki , Y. Kiyohara , H. Uhara , et al., “Cytokine Biomarkers to Predict Antitumor Responses to Nivolumab Suggested in a Phase 2 Study for Advanced Melanoma,” Cancer Science 108, no. 5 (2017): 1022–1031. 28266140 10.1111/cas.13226 PMC5448619

